# Human papillomavirus: footprints in the population of western India

**DOI:** 10.4178/epih.e2021013

**Published:** 2021-02-03

**Authors:** Ashi Robert Thobias, Kinjal Ankit Patel, Supreet Dhananjay Bhatt, Kruti Ashvinkumar Mehta, Chetana Deepal Parekh, Pariseema Sharad Dave, Prabhudas Shankarbhai Patel

**Affiliations:** 1 Biology Department, Gujarat Cancer and Research Institute, Ahmedabad, India; 2 Gujarat University Ahmedabad, India; 3 Surgical Oncology Department, Gujarat Cancer and Research Institute, Ahmedabad, India; 4 Department of Gynecological Oncology, Gujarat Cancer and Research Institute, Ahmedabad, India

**Keywords:** Oropharyngeal neoplasms, Oral neoplasms, Cervical neoplasms, Human papilloma virus

## Abstract

**OBJECTIVES:**

Cancer is a multi-factorial disease, with various intrinsic and environmental factors contributing to its occurrence. Human papillomavirus (HPV) has been associated with the occurrence of many cancers. India severely suffers from 3 HPV-associated cancers (cervical cancer, oral cancer, and oropharyngeal cancer). Hence, the present study aimed to evaluate the HPV burden in these 3 cancers among patients from the western region of India.

**METHODS:**

DNA was isolated from samples from 400 cervical cancer, 127 oral cancer, and 75 oropharyngeal cancer patients. Polymerase chain reaction was performed using degenerate primers for HPV infection.

**RESULTS:**

Overall, HPV infection was observed in 87% of cervical cancer cases, 12.5% of oral cancer cases, and 26.7% of oropharyngeal cancer cases when analyzed with a cumulative detection method using the MY 09/11, GP 5+/6+, and CP I/II primer sets.

**CONCLUSIONS:**

A significant prevalence of HPV infection was detected in all 3 cancers using the degenerate primer sets. This finding implies that testing for HPV infection using multiple primer sets is crucial for determining its actual prevalence in various malignancies.

## INTRODUCTION

Human papillomavirus (HPV) has been identified as an etiological factor for cancer that contributes to 600,000 cancer cases every year worldwide [1]. HPV has been associated with cancer since the 1970s, when the scientist Zur Hausen discovered the presence of HPV in genital warts and cervical cancer [2]. Subsequently, persistent HPV infection has been documented in patients with cervical, vaginal, penile, vulvar, anal, rectal, and oropharyngeal cancer [3,4]. HPV-associated cancers are known to show different clinical, pathological, molecular, and epidemiological profiles as compared to their counterparts. They also have a varied response to therapy and show an overall better survival rate [5-7]. Thus, it is imperative to study the prevalence of these cancers. A standard protocol for the detection of HPV has not yet been defined, which has generated doubts about when and why to assess HPV status [8]. Most studies on the prevalence of HPV have focused on the highly prevalent high-risk human papillomavirus (HR-HPV) types 16 and 18. However, other HPV types also show oncogenic potential and should be evaluated. Furthermore, regional variation has also been observed in HPV prevalence [9].

Degenerate primer sets, including MY 09/11 and GP 5+/6+, are specifically designed in tandem with the highly conserved L1 region of the HPV genome, whereas CP I/II is designed for the E1 region. The degenerate primers were shown to have an improved HPV detection rate in our previous study [10]. Even though the prevalence of HPV-associated cancers, such as cervical cancer, is high in India, countrywide data on HPV infection and genotype distribution are not available, although this information would be useful for a wider vaccination program [11]. Furthermore, there has been an increase of HPV-positive cases of head and neck cancers in the past few decades [12-14]. These cancers are among the major malignancies ailing India [15]. Moreover, there is a dearth of evidence regarding HPV prevalence in western India.

Hence the present study aimed to evaluate the prevalence of HPV in cervical cancer, oral cancer, and oropharyngeal cancer patients in the western region of India. Degenerate primer sets were used for HPV detection, with the specific aim of detecting HPV infection of any strain.

## MATERIALS AND METHODS

The present study evaluated the burden of HPV infection in the 3 most prevalent HPV-associated cancers (i.e., cervical cancer, oral cancer, and oropharyngeal cancer) using degenerate primer sets.

### Sample collection

The patients were enrolled in the study after histopathological confirmation from the pathology department at the Gujarat Cancer and Research Institute, Ahmedabad. Tissue biopsy samples of 400 cervical cancer, 127 oral cancer, and 75 oropharyngeal cancer patients were collected for the study after informed consent. The patients did not undergo any prior treatment for the malignancy. Patients with a simultaneous diagnosis of other major illness or coinfections with human immunodeficiency virus, hepatitis C virus, and hepatitis B virus were excluded from the study. Immediately after collection, the tissue samples were washed with phosphate-buffered saline (pH 7.4) and promptly stored at -80°C until analysis.

### Detection of human papillomavirus infection

DNA was isolated from tissues using a commercially available DNA isolation kit (Qiagen, Valencia, CA, USA). The DNA yield was checked by spectrophotometric analysis (Shimadzu UV-1800, Shimadzu Inc., Kyoto, Japan) and DNA integrity was checked by agarose gel electrophoresis using an 0.8% gel.

The DNA was amplified using the degenerate primer sets MY 09/11, GP 5+/6+, and CP I/II separately on a thermal cycler (Proflex PCR System, Life Technologies, Carlsbad, CA, USA). The polymerase chain reaction (PCR) was prepared using a previously standardized protocol [10].

For the MY 09/11 primers, PCR was carried out as follows: initial denaturation at 95°C for 3 minutes, followed by 40 cycles of denaturation at 95°C for 1 minute, annealing at 49.3°C for 1 minute, and extension at 72°C for 1 minute. In the final cycle, extension was carried out for 10 minutes. For the GP 5+/6+ primers, PCR was carried out as follows: initial denaturation at 95°C for 3 minutes, followed by 40 cycles of denaturation at 95°C for 1 minute, annealing at 48°C for 1 minute, and extension at 72°C for 1 minute. In the final cycle, extension was carried out for 10 minutes. For the CP I/II primers, PCR was carried out as follows: initial denaturation at 95°C for 3 minutes, followed by 40 cycles of denaturation at 95°C for 1 minute, annealing at 56.5°C for 1 minute, and extension at 72°C for 1 minute. In the final cycle, extension was carried out for 10 minutes. The samples were checked for the presence of HPV infection using 2% agarose gel electrophoresis.

The MY 09/11 and GP 5+/6+ primers are designed to detect the L1 region of a wide range of the HPV genome, while CP I/II detects the E1 region (Table 1). A combination of these primer sets may curb the rate of false negativity for HPV infection.

### Ethics statement

The study was approved by the Gujarat Cancer and Research Institute/the Gujarat Cancer Society Ethics Committee (approval no. EC/31/2018). Informed consent was confirmed by the committee.

## RESULTS

### Clinicopathological features of the study cohort

The present study was carried out in 3 highly prevalent HPV-associated cancers (i.e., cervical cancer, oral cancer, and oropharyngeal cancer). The clinical and clinicopathological parameters of the cervical cancer patients (n= 400), oral cancer patients (n= 127), and oropharyngeal cancer patients (n= 75) are given in Tables 2-4, respectively.

### Prevalence of human papillomavirus infection by degenerate primer sets

**Figure 1.**
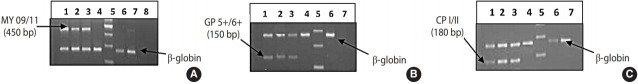
Representative patterns of gel electrophoresis of polymerase chain reaction by degenerative primers (A) MY 09/11, (B) GP 5+/6+, and (C) CP I/II.

All 3 cohorts were screened for comprehensive HPV infections using the degenerate primer sets MY 09/11, GP 5+/6+ and CP I/II. Representative PCR patterns are depicted in Figure 1.

### Cervical cancer

**Figure 2.**
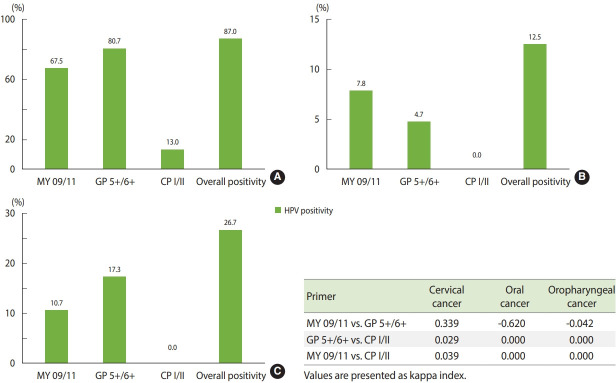
Human papillomavirus (HPV) positivity by degenerative primer sets in (A) cervical cancer, (B) oral cancer, and (C) oropharyngeal cancer.

Out of the cervical cancer patients enrolled in the study, 67.5% were positive for HPV infection by the MY 09/11 primer set, 80.7% of cases were positive by the GP 5+/6+ primer set, and 13.0% of cases were positive by the CP I/II primer set (Figure 2A). The cumulative HPV infection rate detected in the cohort by the combination of all 3 degenerate primer sets was 87.0% (Figure 2A). There was marginal agreement between the detection of HPV by the MY 09/11 and GP 5+/6+ primer sets (kappa index= 0.339), whereas the MY 09/11 and CP I/II primer sets (kappa index= 0.039) and the GP 5+/6+ and CP I/II primer sets (kappa index= 0.029) were not in agreement (Figure 2).

### Oral cancer

In the investigation of 127 oral cancer cases, we observed that 7.8% of cases were positive for HPV infection by MY 09/11, while 4.7% of cases were positive by GP 5+/6+ and all cases were negative for HPV infection by the CP I/II primer set (Figure 2B). The total HPV positivity rate according to the combined investigation using the degenerate primer sets was 12.5% (Figure 2B). There was no agreement in the detection of HPV between the MY 09/11 and GP 5+/6+ primer sets (kappa index= -0.620), between the MY 09/11 and CP I/II primer sets (kappa index= 0.000), and between the GP 5+/6+ and CP I/II primer sets (kappa index= 0.000) (Figure 2).

### Oropharyngeal cancer

Among 75 oropharyngeal cancer cases, HPV positivity was found in 10.7% of cases using the MY 09/11 primer set and in 17.3% of cases using the GP 5+/6+ primers. Similar to oral cancer, no positivity was observed using the CP I/II primer set (Figure 2C). A total HPV positivity rate of 26.7% was observed in the cohort (Figure 2C). There was no agreement in the detection of HPV between the MY 09/11 and GP 5+/6+ primer sets (kappa index= -0.042), between the MY 09/11 and CP I/II primer sets (kappa index= 0.000), and between the GP 5+/6+ and CP I/II primer sets (kappa index=0.000) (Figure 2).

## DISCUSSION

The present study evaluated the burden of HPV infection in the 3 most prevalent HPV-associated cancers (i.e., cervical cancer, oral cancer, and oropharyngeal cancer). The study was carried out in patients from the western region of India using degenerate primer sets. Our study demonstrated a comprehensive screening module to detect HPV infections without exclusion of any HPV type.

We observed that 67.5% of cervical cancer cases were positive for HPV infection using the MY 09/11 primer set. Similar results were obtained in a study conducted in Odisha, which showed a 60.3% infection rate when analyzed using the PGMY 09/11 primer sets [11]. In contrast, a multi-center study from India showed HPV infection in 83.4% of cervical cancer specimens [16]. In this cohort, 80.7% of cases were positive using the GP 5+/6+ primer set. A study from central India reported that 93.3% of cases were positive using the GP 5+/6+ primer set [17], while a study conducted in Karachi, Pakistan showed only a 27.3% prevalence rate in females with cervical abnormalities [18]. When analyzed with the CP I/II primer set, 13% of cases were positive for infection, while the total positivity observed with respect to all 3 degenerate primer sets was 87.0%, which is higher than was reported in Tamil Nadu using the MY 09/11 and GP 5+/6+ primer sets in a study that found an overall HPV infection rate of 54.9% [19]. Moreover, studies from India have reported varying prevalence rates (15-85%) of HPV infection in cervical cancer patients [20].

In the investigation of 127 oral cancer cases, we observed that 7.8% of cases were positive for HPV infection by the MY 09/11 primer set, while 4.7% of cases were positive by the GP 5+/6+ primer set. This rate is lower than that reported in another study that recorded an HPV prevalence of 35.8% in oral mucosa samples when analyzed with the GP 5+/6+ primer set, whereas a positivity rate of only 2.2% was reported using the MY 09/11 primer set [21]. Furthermore, studies from south and southwest India showed no presence of HPV infection [22,23]. All the cases were negative for HPV infection by the CP I/II primer sets. The total HPV positivity by the combined investigation using degenerate primer sets was 12.5%. This rate is lower than that reported for the northeast region of India, where HR-HPV infection was observed to be present in 27.9% in oral cancer patients [24]. This may be attributed to the loco-regional variance in HPV prevalence.

We also investigated the presence of HPV infection in 75 oropharyngeal cancer cases and observed HPV positivity in 10.7% of cases by the MY 09/11 primer set and 17.3% of cases by the GP 5+/6+ primer set. Similar to oral cancer, no positivity was observed using the CP I/II primer set. A study conducted in Delhi reported a 22.8% HPV infection rate in oropharyngeal cancer cases when analyzed using consensus primers [25]. A total HPV positivity rate of 26.7% was observed in the cohort. A study conducted in the United States reported that 70% of cases of oropharyngeal cancers were attributable to HPV infection [26], while a study in Karnataka reported no prevalence of HPV in oropharyngeal cancers [27]. HPV prevalence was observed to be low in both oral and oropharyngeal cancer patients from the western region of India, as the relatively high rate of incidence for both these cancers is attributable to the high consumption of tobacco in this region. Hence, the majority of cancer cases result from tobacco-related oncogenesis rather than HPV infection [28,29].

The study demonstrated a high rate of HPV prevalence (87.0%) in cervical cancer patients. Moreover, we also observed a notable HPV prevalence (12.5 and 26.7%) in oral and oropharyngeal cancers, respectively. HPV-positive cancers represent a distinct molecular and clinical entity from HPV-negative cancers [26,27,30,31]. Furthermore, separate guidelines have been added for the staging of HPV-positive oropharyngeal cancers in the eighth edition of the American Joint Committee on Cancer cancer staging guidelines. These guidelines are anticipated to provide a much more accurate and reasonable prediction of survival for newly diagnosed patients [32]. Furthermore, the kappa index between the primer sets was very low, showing no agreement between the primer sets. Thus, it is imperative to screen the population using all 3 primer sets for actual detection. Furthermore, it is especially important to study the HPV burden in HPV-associated cancers with a focus on HR-HPV types. This will help improve the differentiation of HPV-associated cancers and result in a better diagnostic approach for disease management. The screening module with degenerate primer sets (MY 09/11, GP 5+/6+ and CP I/II) used in the present study will aid in screening a higher rate of HR-HPV infections.

In conclusion, the present investigation provides vital data for the study of the burden of HPV-associated cancers. The study found a high rate of HPV infection in cervical cancer and a notable prevalence of HPV infection in oral and oropharyngeal cancers using degenerate primers. Thus, the present study demonstrates the use of a comprehensive screening module for the effective detection of HPV infection in these 3 major malignancies without bias towards any HPV type.

## Figures and Tables

**Table 1.** Primer sequence, amplicon size and region specificity of degenerative primer sets

Variables	MY 09/11	GP 5+/6+	CP I/II
Sequence			
Forward	5’-CGT CCM ARR GGA WAC TGA TC-3’	5’-TTT GTT ACT GTG GTA GAT ACT AC-3’	5’-TTA TCW TAT GCC CAY TGT ACC AT-3’
Reverse	5’-GCM CAG GGW CAT AAY AAT GG-3’	5’-GAA AAA TAA ACT GTA AAT CAT ATT C-3’	5’-ATG TTA ATW SAG CCW CCA AAA TT-3’
Amplicon size (bp)	450	150	180
HPV region	L1	L1	E1

HPV, human papillomavirus.

**Table 2.** Clinical and clinicopathological details of the cervical cancer cohort

Characteristics	No. of patients (%)
Age (yr)	
<50	233 (58.3)
≥50	167 (41.8)
Menopause status	
Premenopausal	90 (22.5)
Postmenopausal	185 (46.3)
Unknown	125 (31.2)
Histology	
Squamous cell carcinoma	365 (91.2)
Adenocarcinoma	30 (7.5)
Unknown	5 (1.2)
Type of growth	
Exophytic	103 (25.7)
Infiltrative	94 (23.5)
Unknown	203 (50.7)
Differentiation	
Well	14 (3.5)
Moderate	254 (63.5)
Poor	75 (18.7)
Unknown	57 (14.2)
Stage	
I	31 (7.8)
II	62 (15.5)
III	234 (58.5)
IV	11 (2.7)
Unknown	62 (15.5)
Recurrence	
Yes	16 (4.0)
No	212 (53.0)
Unknown	172 (43.0)

**Table 3.** Clinical and clinicopathological details of the oral cancer cohort

Characteristics	No. of patients (%)
Age (yr)	
<45	59 (46.5)
≥45	68 (53.5)
Sex	
Male	105 (82.7)
Female	22 (17.3)
Site of origin	
Buccal	48 (37.8)
Tongue	43 (33.8)
Other	18 (14.2)
Mixed	18 (14.2)
Tobacco habit	
Yes	106 (83.46)
No	21 (16.54)
Histology	
Squamous cell carcinoma	124 (97.6)
Verrucous carcinoma	2 (1.6)
Unknown	1 (0.8)
Differentiation	
Well	37 (29.1)
Moderate	78 (61.4)
Poor	5 (3.9)
Unknown	7 (5.6)
Stage	
I	13 (10.2)
II	45 (35.4)
III	13 (10.2)
IV	51 (40.2)
Unknown	5 (4.0)
Lymph node metastasis	
Yes	39 (30.7)
No	84 (66.1)
Unknown	4 (3.2)

**Table 4.** Clinical and clinicopathological details of the oropharyngeal cancer cohort

Characteristics	No. of patients (%)
Age (yr)	
<57	38 (50.6)
≥57	37 (49.3)
Sex	
Male	74 (98.6)
Female	1 (1.4)
Site of initiation	
Base of tongue	30 (40.0)
Valeculla	4 (5.3)
Tonsillar fossa	10 (13.3)
Mixed site	5 (6.7)
Other site	26 (34.7)
Tobacco habit	
Yes	66 (88.0)
No	1 (1.3)
Unknown	8 (10.7)
Histology	
Squamous cell carcinoma	66 (88.0)
Unknown	9 (12.0)
Tumor differentiation	
Well	6 (8.0)
Moderate	42 (56.0)
Poor	11 (14.6)
Unknown	16 (21.4)
